# Unveil *cis*-acting combinatorial mRNA motifs by interpreting deep neural network

**DOI:** 10.1093/bioinformatics/btae262

**Published:** 2024-06-28

**Authors:** Xiaocheng Zeng, Zheng Wei, Qixiu Du, Jiaqi Li, Zhen Xie, Xiaowo Wang

**Affiliations:** Ministry of Education Key Laboratory of Bioinformatics; Center for Synthetic and Systems Biology; Bioinformatics Division, Beijing National Research Center for Information Science and Technology; Department of Automation, Tsinghua University, Beijing, 100084, China; Ministry of Education Key Laboratory of Bioinformatics; Center for Synthetic and Systems Biology; Bioinformatics Division, Beijing National Research Center for Information Science and Technology; Department of Automation, Tsinghua University, Beijing, 100084, China; Ministry of Education Key Laboratory of Bioinformatics; Center for Synthetic and Systems Biology; Bioinformatics Division, Beijing National Research Center for Information Science and Technology; Department of Automation, Tsinghua University, Beijing, 100084, China; Ministry of Education Key Laboratory of Bioinformatics; Center for Synthetic and Systems Biology; Bioinformatics Division, Beijing National Research Center for Information Science and Technology; Department of Automation, Tsinghua University, Beijing, 100084, China; Ministry of Education Key Laboratory of Bioinformatics; Center for Synthetic and Systems Biology; Bioinformatics Division, Beijing National Research Center for Information Science and Technology; Department of Automation, Tsinghua University, Beijing, 100084, China; Ministry of Education Key Laboratory of Bioinformatics; Center for Synthetic and Systems Biology; Bioinformatics Division, Beijing National Research Center for Information Science and Technology; Department of Automation, Tsinghua University, Beijing, 100084, China

## Abstract

**Summary:**

*Cis*-acting mRNA elements play a key role in the regulation of mRNA stability and translation efficiency. Revealing the interactions of these elements and their impact plays a crucial role in understanding the regulation of the mRNA translation process, which supports the development of mRNA-based medicine or vaccines. Deep neural networks (DNN) can learn complex *cis*-regulatory codes from RNA sequences. However, extracting these *cis*-regulatory codes efficiently from DNN remains a significant challenge. Here, we propose a method based on our toolkit NeuronMotif and motif mutagenesis, which not only enables the discovery of diverse and high-quality motifs but also efficiently reveals motif interactions. By interpreting deep-learning models, we have discovered several crucial motifs that impact mRNA translation efficiency and stability, as well as some unknown motifs or motif syntax, offering novel insights for biologists. Furthermore, we note that it is challenging to enrich motif syntax in datasets composed of randomly generated sequences, and they may not contain sufficient biological signals.

**Availability and implementation:**

The source code and data used to produce the results and analyses presented in this manuscript are available from GitHub (https://github.com/WangLabTHU/combmotif)

## 1 Introduction

Messenger RNA (mRNA) is a transcript of a gene, which carries the genetic information from the cell nucleus to be translated into protein by ribosomes. The translation process of mRNA is regulated by RNA binding proteins (RBPs) (Gerstberger *et al.* 2014), MicroRNAs (Bushati and Cohen 2007), and other molecules in the cytoplasm (Vitreschak *et al.* 2004). These molecules bind corporately to specific RNA motif patterns with higher affinity or special secondary structure (Leppek *et al.* 2018). Correspondingly, the sequence motifs and their arrangements, i.e. motif number, order, orientation, and spacing (termed here collectively “motif syntax”) determine the regulation of protein, including protein expression level, degradation rate, ribosome loading level and other factors (Pique *et al.* 2008, Cheng *et al.* 2017, Sample *et al.* 2019, Agarwal and Kelley 2022, de Almeida *et al.* 2022). Therefore, discovering RNA motifs and their syntax is vital for a more comprehensive understanding of mRNA translation process, advancing the development of mRNA-based medicines or vaccines (Zeng *et al.* 2020).

**Figure 1.**
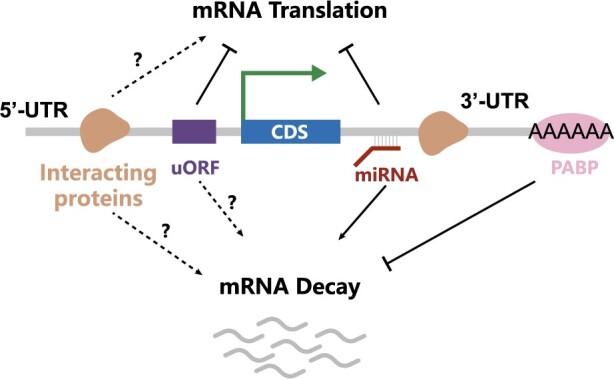
Crucial factors influencing mRNA translation efficiency and stability: MicroRNAs, RNA Binding Proteins, uORF, PolyA tail, etc.

The *cis*-acting regulatory region of mRNA mainly consists of 5ʹcap, 5ʹ untranslated region (5ʹUTR), open reading frame (ORF) sequence, 3ʹ untranslated region (3ʹUTR) (Fig. 1). The components collectively influence both the expression level and decay rate of mRNA (Pardi *et al.* 2018). 5ʹUTRs and 3ʹUTRs can be bound by RBPs and MicroRNAs (Mignone *et al.* 2002, Lim *et al.* 2005), which play the key role in the *cis*-acting regulation of the translation and degradation process (Chatterjee and Pal 2009).

Various biological experiments have been designed to discover RBPs and MicroRNAs binding motifs. Typically, immunoprecipitation-based technologies are commonly used to identify RBP targets and binding sites across the transcriptome (Ramanathan *et al.* 2019). However, these approaches entail high experimental costs, lengthy timelines, and significant technical challenges (Oikonomou *et al.* 2014). Furthermore, these methods are limited to a single motif. Discovering relevant motif combinations remains challenging.

In recent years, convolutional neural networks (CNN) have shown successful applications in predicting mRNA functions, outperforming traditional methods that based on feature engineering (Jaganathan *et al.* 2019, Sample *et al.* 2019, Agarwal and Kelley 2022), indicating CNNs’ ability in deciphering motifs and complex regulatory grammars in mRNA sequences. However, CNNs are black boxes that make it difficult for researchers to interpret the motifs and their combinations learned by models. In current exploitation of CNNs for uncovering biological insights, limited high-quality and quantity data for training high-performance CNNs and inefficient interpretation methods present challenges. To address these issues, many researchers have built deep-learning models based on artificial datasets or endogenous datasets and utilized interpretation methods such as filter visualization (Alipanahi *et al.* 2015) or TF-MoDISco (Shrikumar *et al.* 2018) to discover key elements influencing mRNA translation efficiency and stability (Sample *et al.* 2019, Agarwal and Kelley 2022, Zheng *et al.* 2023).

However, there are some common limitations in related works. First of all, the interpretation methods in existing works do not solve the multifaceted neuron problem (Nguyen *et al.* 2016, Goh *et al.* 2021, Wei *et al.* 2023), resulting in low-quality and less diverse motifs in the interpretations. Besides, the existing works lack the investigation into motif syntax learned by CNN, which is crucial for the regulation of mRNA stability and translation efficiency (Pique *et al.* 2008, Cheng *et al.* 2017, de Almeida *et al.* 2022). In addition, the poor quality of training data for CNN is leading to the model’s inability to learn effective signals. Developing effective approaches to systematically analyze the influence of motif syntax on mRNA translation efficiency and stability is essential.

In this paper, we proposed a comprehensive pipeline tailored specifically for the interpretation of RNA-sequence-to-function models, incorporating motif discovery, motif contribution analysis, and motif interaction analysis. Our pipeline exhibits compatibility with state-of-the-art CNN models used across various domains. A significant advantage of using our pipeline lies in its capacity to yield dependable insights into the motifs and their interactions that neural networks have learned from biological data.

The key component of our pipeline is NeuronMotif (Wei *et al.* 2023), our recently proposed interpretation method designed to decipher the mixture of motif patterns in the deeper layers of CNN. NeuronMotif has been successfully applied to interpret *cis*-regulatory DNA sequences across a range of deep neural network models. In this study, we applied NeuronMotif to unveil *cis*-acting mRNA motifs, and the motifs discovered by NeuronMotif demonstrate a greater diversity and higher quality compared to those discovered by existing methods. Besides, we extended NeuronMotif with in silico mutagenesis (ISM) (Zhou and Troyanskaya 2015, Kim *et al.* 2021) to enable the investigation of the impact of key motifs or motif combinations on the model’s prediction. Our results suggested that over 50% of the motif pairs discovered by NeuronMotif exhibit significant interactions. We have also proved that the utilization of our method enables the cost-effective acquisition of crucial regulatory codes in mRNA translation. In addition, we carried out simulation experiments. The results revealed that CNN can hardly learn meaningful motif interactions from datasets composed of randomly generated sequences. This suggests that it is more effective to choose endogenous sequences or artificially designed sequences as the training data for CNN.

## 2 Materials and methods

### 2.1 Dataset

Our study utilized two datasets: one focused on mRNA half-life and the other on mRNA translation efficiency.

Agarwal and Kelley (2022) established a compendium about mRNA half-life encompassing 39 human transcriptomes. In total, they provide a debiased half-life dataset of 13 000 mRNA sequences, consisting of 5ʹUTR, CDS, and 3ʹUTR.

As Mean Ribosome Load (MRL) can serve as an indicator of translation efficiency, we also used the dataset proposed by Sample *et al.* (2019), including a collection of 280 000 random 5ʹ UTR sequences and the MRL levels of the corresponding mRNA transfected into HEK293T cells.

### 2.2 Deep learning-based motif interpretation

#### 2.2.1 Model for half-life and MRL prediction

**Figure 2.**
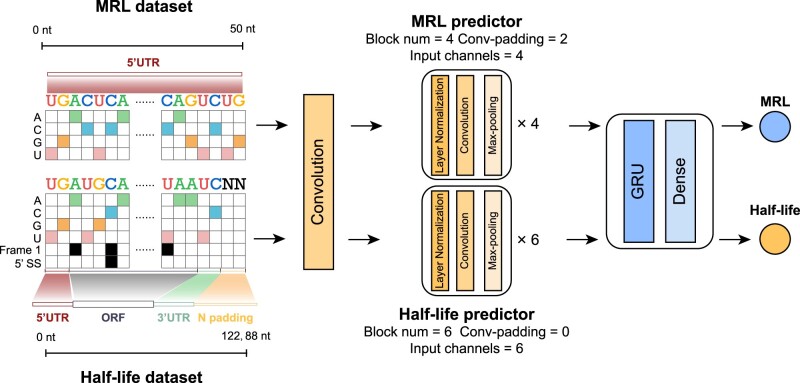
A hybrid convolutional and GRU neural network to predict half-life and MRL from 5ʹUTR or complete mRNA sequence. The MRL predictor and the half-life predictor have a similar architecture, the only difference between them being the number of intermediate blocks, which are 4 and 6, respectively. They are trained on the MRL dataset and half-life dataset separately. Besides, the half-life dataset includes additional encoding of the first frame of each codon and 5ʹ splice site junctions.

We applied the same architecture as Saluki model (Agarwal and Kelley 2022) for both half-life and MRL prediction, which is a hybrid convolutional and recurrent deep neural network (Fig. 2). Here, we referred to these two models as the half-life predictor and MRL predictor respectively. The MRL predictor and the half-life predictor have a similar architecture, the only difference between them being the number of intermediate blocks, which are 4 and 6, respectively. The intermediate blocks are composed of layer normalization layers, convolutional layers and max-pooling layers, followed by a gated recurrent unit (GRU), and a densely connected layer. The MRL predictor and the half-life predictor are trained on the MRL dataset and half-life dataset respectively.

#### 2.2.2 Motif discovery

We applied NeuronMotif (Wei *et al.* 2023) to discover motifs and motif combinations learned by neural network. NeuronMotif is a filter visualization and clustering based interpretation method, which is specifically designed for deep-learning interpretation in the genome field.

To ascertain which query motif exhibit significant similarity to annotated RBP binding motifs or MicroRNA binding motifs, a motif similarity analysis was conducted using Tomtom from the meme-suite toolkit version 5.5.3. RBP binding motifs are annotated by RNAcompete (Ray *et al.* 2013) and MicroRNA motifs are annotated by miRBase (Griffiths-Jones *et al.* 2006). Only motif pairs meeting the criterion of *q*-value ≤ 0.001 will be considered as significantly similar.

#### 2.2.3 Motif contribution analysis

Although we have obtained motifs learned by CNN, it is necessary to determine whether these motifs have a positive or negative contribution on mRNA stability or translation efficiency. In addition, the effects of individual motifs will contribute to our subsequent analysis of motif interactions. Here, we proposed a simple method to analyze motif contribution.

For a specific neuron (CNN filter), we divide the dataset into two groups based on whether the samples activate it or not. Then, we conduct a statistical significance test to compare the means of the mRNA properties from the two groups of samples. If the mean of the group that activates the neuron is significantly higher than the other group, it indicates that the visualized motif from this neuron has an enhancing effect on MRL. Otherwise, this neuron has an inhibitory effect on MRL.

#### 2.2.4 Motif interaction analysis

**Figure 3.**
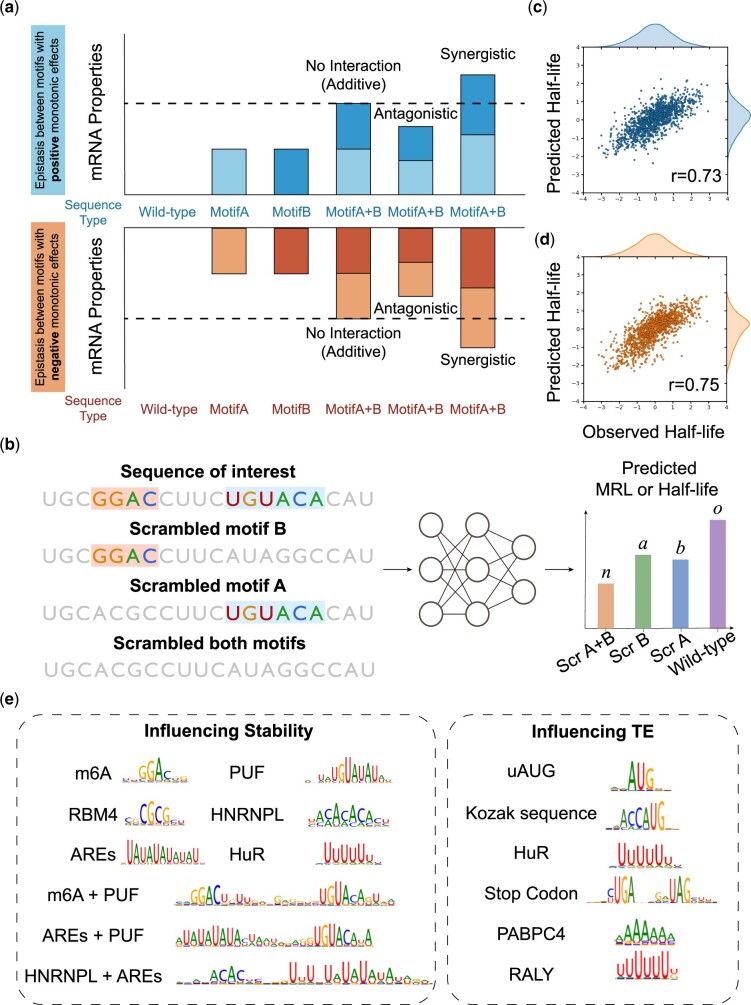
(a) The illustration of three types of epistasis interaction. (b) Motif mutagenesis analysis. (c) The performance on the human test set of half-life predictor trained on human gene sequences. The scatter plot of predicted half-life and observed half-life and the person correlation coefficient *r* = 0.73. (d) The performance on the human test set of half-life predictor trained on both human and mouse gene sequences. (e) The motifs and motif combinations interpreted from half-life predictor and MRL predictor by NeuronMotif. The motifs learned by half-life predictor will influence mRNA stability, and motifs learned by the MRL predictor will influence mRNA translation efficiency. TE: Translation Efficiency.

The combinations of motifs that appear simultaneously in mRNA sequences may have different types of quantitative joint effect on mRNA translation efficiency and stability. We primarily consider three types of joint effect, namely no epistasis, synergistic epistasis and antagonistic epistasis (Fig. 3a).

We utilized a combinatorial in silico motif mutagenesis approach (Kim *et al.* 2021) to assess epistatic interactions between pairs of candidate motif instances (denoted as A and B) within a sequence. We represent model predictions as *o* for intact sequence (containing both A and B), *b* for Motif A scrambled, *a* for Motif B scrambled, and *n* for the null sequence (both A and B scrambled) (Fig. 3b). Referring to the epistasis analysis method (Kim *et al.* 2021, Moore and Williams 2016), we compare the joint effect size (o−n) to the sum of the marginal effect sizes (a − n) + (b − n)=(a + b − n) by conducting a Wilcoxon signed rank test on the paired values (joint versus sum of marginals) for all instances of a motif pair. When (o − n) is significantly >(a + b − n), there is a synergistic interaction between Motif A and Motif B. When (o − n) is significantly smaller than (a + b − n), there is an antagonistic interaction between Motif A and Motif B. In cases where there is no significant difference, Motif A and Motif B are considered to be independent and do not influence each other, indicating an additive relationship. We summarized the interaction judgement rules in Table 1.

**Table 1.** Judgment rules for interaction.^a^

Motif A	Motif B	Condition	Interaction
Positive	Positive	(o−n)>(a−n)+(b−n)	Syn
Positive	Positive	(o−n)<(a−n)+(b−n)	Ant
Negative	Negative	(o−n)>(a−n)+(b−n)	Ant
Negative	Negative	(o−n)<(a−n)+(b−n)	Syn
P/N	P/N	(o−n)=(a−n)+(b−n)	Add
Positive	Negative	(o−n)>(a−n)	Sign
Positive	Negative	(o−n)<(b−n)	Sign

a Syn, synergistic; Ant, antagonistic; Add, additive.

It should be noted that above rules are effective when both Motif A and Motif B have positive or negative effects on mRNA translation efficiency or stability (Moore and Williams 2016). When Motif A and Motif B exhibit opposing effects, we propose a rule that applies to this scenario. Assuming that Motif A has a positive effect (a > n), and Motif B has a negative effect (b < n), if (o − n) is significantly >(a − n), this indicates a sign epistatic interaction (Weinreich *et al.* 2005) between Motif A and Motif B. Conversely, if (o − n) is significantly smaller than (b − n), this also indicates a sign epistatic interaction.

## 3 Results

### 3.1 NeuronMotif outperforms existing interpretation methods on motif discovery

**Figure 4.**
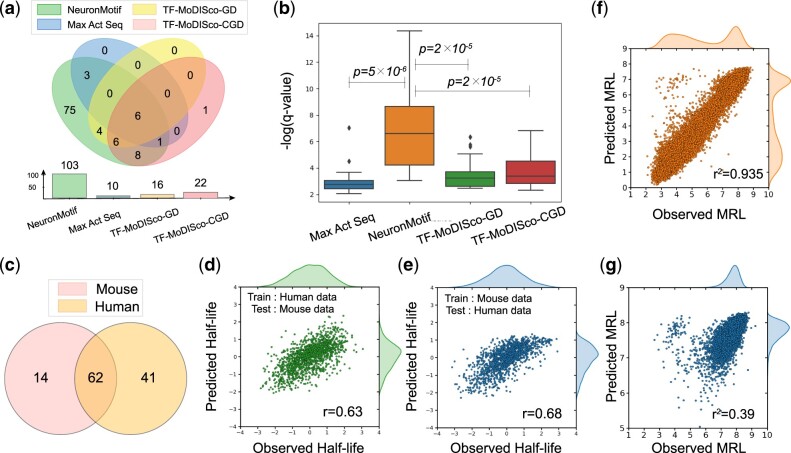
(a) This Venn diagram illustrates the diversity of query motifs obtained by interpreting the model using four interpretation methods, namely NeuronMotif, Maximal activation seqlet, TF-MoDISco-GD, and TF-MoDISco-CGD, respectively. (b) The boxplot illustrates the comparison of query motif quality obtained by interpreting the model using four interpretation methods. The quality metric is the similarity between the query motif and the database, measured by the *q*-value. A smaller *q*-value indicates a higher similarity. (c) The Venn of the motifs learned by half-life predictors trained on human and mouse dataset, respectively. (d) Predictor trained on human train set could explain 63% of half-life variation in the mouse test set. (e) Predictor trained on mouse train set could explain 68% of half-life variation in the human test set. (f) The performance of MRL predictor trained on AUG dataset. (g) The performance of MRL predictor trained on uAUG-free dataset.

An accurate deep-learning model is crucial for discovering motifs and motif combinations. We trained two deep-learning models (Fig. 2) based on the half-life dataset and MRL dataset respectively. The half-life predictor’s final performance is similar to the Saluki model (Agarwal and Kelley 2022), with a slightly inferior Pearson correlation coefficient of 0.73 since mouse data are excluded (Fig. 3c). Our MRL predictor ($R^{2}=0.935$) performs (Fig. 4f) as well as Optimus 5-Prime (Sample *et al.* 2019) ($R^{2}=0.934$).

We used NeuronMotif, TF-MoDISco (Shrikumar *et al.* 2018) with saliency map (Simonyan *et al.* 2013), TF-MoDISco with Corrected Gradient (Majdandzic *et al.* 2023), and Maximal Activation Seqlet (Alipanahi *et al.* 2015), to interpret the half-life predictor and MRL predictor. Then, we compared their interpretation results from two perspectives: motif diversity and motif quality. TF-MoDISco is a versatile tool capable of accepting various attributions. For our analysis, we treat saliency map (gradients) and corrected gradients as attributions, respectively, hereafter denoted as TF-MoDISco-GD and TF-MoDISco-CGD. We only presented the interpretation results of the half-life predictor, and the results of the MRL predictor have been placed in our github repository.

As for motif diversity, we collected the motifs found by each interpretation method and applied a Venn diagram to illustrate the motif diversity of these methods (Fig. 4a). Our results suggest that NeuronMotif significantly reveals the largest variety of motifs, nearly encompassing all motifs found by the other methods. This indicates that NeuronMotif can extract the patterns learned by the model more comprehensively.

As for motif quality, we measured the quality of the query motif by assessing its similarity to the database motif, and this similarity is quantified by the *q*-value output from TomTom. The lower the *q*-value, the more similar the current query motif is to motifs in the database, indicating higher quality. We selected the intersection of query motifs interpreted by the four methods for analysis. Then, we conducted a Wilcoxon signed rank test on the *q*-values of query motifs from NeuronMotif and the other methods respectively. The quality of query motifs from NeuronMotif is significantly higher than the other methods (Fig. 4b), with *P*-values of $5\;\times \;10^{-6}$ (with Maximal Activation Seqlet), $2\times 10^{-5}$ (with TF-MoDISco-GD), and $2\times 10^{-5}$ (with TF-MoDISco-CGD), respectively.

The above results indicate that NeuronMotif is capable of uncovering diverse and biologically meaningful motifs in mRNA.

### 3.2 Discovered motifs and motif combinations are supported by multiple sources of biological data

Through interpreting the MRL predictor and half-life predictor, we identified motifs and motif combinations that align with prior research and established biological priors (Fig. 3e).

Many of the patterns we found can be supported by literature, corresponding to the motifs founded by Agarwal and Kelley (2022) and Zheng *et al.* (2023). For example, HuR has been proved to bind to 5ʹUTR (Meng *et al.* 2005); AU-rich elements in the 3ʹUTR are the most common determinants of RNA stability in mammalian cells (Chen and Shyu 1995); RMB4 element is known to suppress the cap-dependent initiation process (Lin *et al.* 2007); m6A motif (“GGACU”) were enriched as significant motifs in the 3ʹUTR (Meyer *et al.* 2012). It implies that our interpretation method is effective in interpreting crucial biological motifs in mRNA.

In addition, We conducted a statistical analysis on whether previously discovered motifs have a positive or negative contribution to mRNA stability or translation efficiency. The analysis procedure has been described in the session of “Motif contribution analysis.” In this way, we found some typical motifs that destabilize mRNA. For instance, AREs, PUF element, and m6A motif have been shown to repress stability (Agarwal and Kelley 2022). RBP binding motifs of HuR, RALY and ZC3H14 have positive contributions to mRNA translation efficiency (Zheng *et al.* 2023).

The interaction between motifs may have a significant impact on mRNA translation efficiency and stability (Pique *et al.* 2008, Cheng *et al.* 2017, de Almeida *et al.* 2022). We leveraged NeuronMotif to interpret the neurons in deeper layers of the neural network and found motif combinations successfully. A typical example we found is the combination of the PUF element (“UGUAHAUA”) and the m6A motif (“GGACU”), which has been demonstrated to influence the stability of mRNA (Vaidyanathan *et al.* 2017). While directly applying TF-MoDISco could only find individual PUF element and m6A motif, but not their combinations (Agarwal and Kelley 2022). Therefore, NeuronMotif excels in discovering motif combinations.

### 3.3 Discovered motif combinations are significant in mutagenesis analysis

The mRNA sequence encompasses a variety of motif combinations, however, only a subset of those motif pairs exhibits interaction. Fortunately, NeuronMotif has efficiently screened the most potential motif combinations for us, and there is no need to consider all possible combinations ($n^{2}$, where *n* is the number of motif candidates).

We used an in-silico motif mutagenesis analysis method (Fig. 3b) to quantify the influence of pairs of co-occurring motifs on mRNA half-life. Specifically, for a motif pair A and B, we studied whether the effects of motif A and motif B on model’s output are independent.

**Figure 5.**
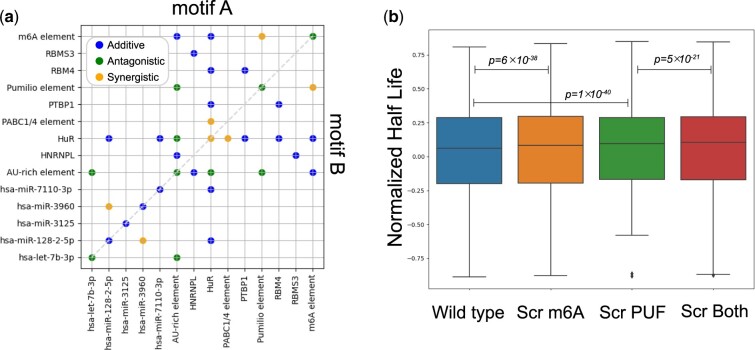
(a) The motif interaction map obtained through motif mutagenesis and wilcoxon signed rank. The dots on the map represent instances where NeuronMotif discovered combinations of corresponding motif pairs. The different colors of these dots indicate that Motif A and Motif B have different types of interactions. All motifs in the interaction map have negative contributions to mRNA stability, thus, there is no case of sign epistasis. (b) Boxplot of the mutagenesis results of m6A motif and PUF element. “Scr m6A” corresponds to the predictions of half-life predictors on sequences whose m6A motif has been scrambled. “Scr Both” corresponds to the predictions on sequences whose m6A motif and PUF element both have been scrambled.

Through our method, we discovered that there is interaction between the PUF element and m6A motif (Vaidyanathan *et al.* 2017). We conducted the Wilcoxon signed rank test (*P*-value=0.0048) on the paired values (joint versus sum of marginals) for all instances containing both PUF element and m6A motif (278 sequences in total) (Fig. 5b). Both PUF element and m6A motif will reduce mRNA stability and the co-occurrence of them will significantly further reduce mRNA stability.

We generated an interaction map to illustrate the interactions between motifs (Fig. 5a). In the interaction map, the absence of points represents motif pairs for which the combination was not found in the interpretation results. The blue dots represent motif pairs (Motif A and Motif B) that are independent of each other. Although the neural network suggests that there should be an interaction between these two motifs, the statistical test results do not show an interaction. This discrepancy may arise from the noise in the dataset on one hand and insufficient data for the mutagenesis statistical test on the other. In summary, NeuronMotif identified 39 motif combinations, and statistical testing confirmed that 17 motif combinations among them exhibit significant interactions.

### 3.4 Randomly generated sequences are inefficient for motif combinations discovery

#### 3.4.1 Randomly generated 5ʹUTR sequences introduce uAUG bias

**Figure 6.**
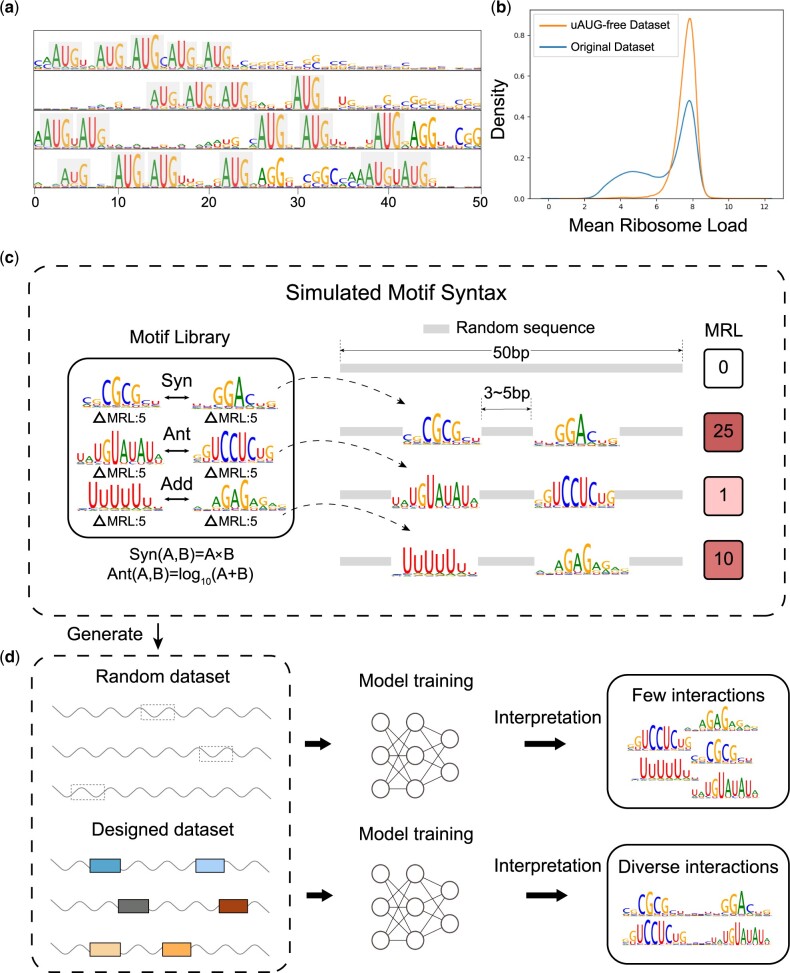
(a) The interpretation results of the conv4 neurons in MRL predictor. Repeat AUGs are the preferred patterns of most neurons in the MRL predictor. (b) The distribution of the MRL in the original dataset and uAUG-free dataset. (c) We created a library of motifs and defined two motif interactions, namely synergistic and antagonistic interaction. (d) The sequences in the random dataset are generated randomly; the sequences in designed dataset are random background sequences embedded with two arbitrary motifs in library. Two deep-learning models are trained on these two datasets, respectively (rand-model and desi-model for simple), followed by interpretation with NeuronMotif. Rand-model only learns individual motifs, while desi-model learns various motif interaction.

uAUG is a shortcut for a model to make predictions. Since the interpretation results primarily display repeated uAUG as the most common motifs (Fig. 6a), MRL predictor model carries significant risk as it heavily relies on the presence of AUG motifs in the sequence to predict MRL. To eliminate the influence of uAUG, we removed sequences containing uAUGs and generated an uAUG-free dataset.

We retrained the MRL predictor on the uAUG-free dataset, while the model’s performance significantly declined: it could only explain 40% of the MRL variation (Fig. 4g). In addition, the probability distribution curve of MRL on the original dataset resembles a mixture of Gaussian distributions (Fig. 6b), and the left peak corresponds to sequences containing AUG motifs. As expected, the left peak disappeared in the distribution curve of the MRL on the uAUG-free dataset, which suggests that AUG is a significant factor influencing MRL. Therefore, uAUG is the dataset bias and we should remove uAUG before training.

#### 3.4.2 Randomly generated 5ʹUTR sequences rarely contain strong motif combinations

Uncovering motifs’ impact on mRNA translation efficiency beyond uAUG is also important. We applied the aforementioned approach to MRL predictor, and our results suggested that our approach successfully discovered functional motifs and identified their contributions to mRNA translation efficiency. However, they can hardly learn the combinations of these motifs.

We speculate that this phenomenon stems from the lack of signals in the dataset. Short motifs in the genome can be perceived as signals, and the functionality of natural mRNA is often achieved through the integration of these weak signals. Since the training set consists of randomly generated sequences, it is challenging to enrich crucial motif combinations, and consequently, the model fails to learn these motif combinations from such a dataset. To validate this hypothesis, we perform a simulation experiment.

We generated two datasets—one with random sequences and the other with manually designed sequences. “Random sequence” means that each nucleotide in the sequence is randomly selected from “A, G, C, T.” “Designed sequence” means that the sequence always contains a pair of motifs, and their positions or spacing between them are indeterminate, while the remaining nucleotides are randomly selected from “A, G, C, T.” To control variables, the size of both datasets is set at 280 000, consistent with the real MPRA 5ʹUTR dataset (Sample *et al.* 2019). The details of simulation datasets can be accessed in Supplementary files. Subsequently, we trained and interpreted two independent 3-layer CNN-GRU models which have the same architecture as the MRL predictor on each dataset (Fig. 6c and d).

As expected, the model based on the designed dataset learned all six interactions with a true positive rate of 100%, while the model trained on the random dataset did not discover any interactions, resulting in a true positive rate of 0%. To demonstrate the robustness of the results, we gradually reduced the size of the designed dataset from 280 000 until it reached 2000. Even though the dataset contains only 2000 samples, the model is still able to learn three pairs of interactions with a true positive rate of 50%.

Therefore, we do not recommend conducting experiments on random sequences. Instead, the focus should be on endogenous sequences or sequences designed by prior knowledge, which are more likely to contain motif syntax.

### 3.5 Cross-species data are helpful for deep-learning models to learning motifs 

mRNA translation data are usually small due to the specific experiment condition. It is difficult for deep CNN to learn critical motif syntax from insufficient datasets so extending the dataset appropriately is essential. Based on the evolution information, the closely related species may share similar regulatory patterns. Hence, it may be possible to extract knowledge from information from a closely related species. We found that mouse data helps improve the performance of the human model (from r=0.73 to r=0.75 on the test set, Fig. 3d). The model trained on the mouse training set could also perform well on the human dataset (r=0.68 on the human test set, Fig. 4e). In addition, by interpreting the models trained on human and mouse data respectively, we found there are many shared motifs between human and mouse (Fig. 4c). Therefore, cross-species data might be helpful in uncovering regulatory codes in the current species when human data are lacking.

## 4 Conclusion

Various *cis*-acting elements in mRNA regulate mRNA’s translation efficiency and stability. Discovering the interactions of these elements and their impact on mRNA translation rapidly at a low cost is a crucial scientific problem. Deep learning provides a rapid way to learn motif syntax. However, extracting these motif syntaxes from neural networks is limited by the efficiency of the data quality, quantity, and interpretation method.

In this paper, we proposed a method based on NeuronMotif and motif mutagenesis, which not only enables the discovery of diverse and high-quality motifs but also efficiently reveals motif interactions. With this method, we systematically analyzed the learned motif syntax of two deep-learning models, the MRL predictor and the half-life predictor. The interpretation results outperform existing methods and are supported by multiple biological sources, including literature and biological data, offering novel insights for biologists. In addition, based on the interpretation result of NeuronMotif and simulation results, we found that randomly generated sequences are prone to introduce bias, and are less informative for the discovery of motifs and motif interactions using deep learning methods. Instead, the experimental data validating the sequences designed by prior knowledge would be more efficient for deep learning models to explore RNA *cis*-regulatory motif syntaxes.

Although we may unveil reliable regulatory motifs and motif combinations using NeuronMotif and motif mutagenesis (Figs 3a and 5a), there is still rich information learned by the model that can be further explored. For example, motif location is another important information for mRNA sequence design. With the Massively Parallel Reporter Assays (MPRAs) results, Sample *et al.* investigated the impact of uAUGs at different positions within the 50-nucleotide window immediately adjacent to the start codon on MRL (Sample *et al.* 2019). Agarwal *et al.* inserted key motif along the full length of each mRNA to investigate the most informative positions along an mRNA that contribute to half-life (Agarwal and Kelley 2022). Furthermore, the aforementioned simulation results suggested that the ability of our method to discover motifs or motif combinations is influenced by various factors, including the density of motifs within sequences, the size of the dataset and the length of the sequences, among others. Additional work can reveal the impact of various factors on discovery capacity. Finally, the interpretation method proposed in this study will offer an effective means of discovering regulatory elements capable of simultaneously enhancing stability and translation efficiency, along with unraveling their underlying mechanisms. This are potentially applicable to facilitate the design of mRNA sequences for practical biological applications.

Overall, we have developed a pipeline tailored for interpreting RNA sequence-to-function models, encompassing motif discovery, motif contribution analysis, and motif interaction analysis. This pipeline is compatible with state-of-the-art CNN models across various domains. A significant advantage of using our pipeline is its ability to provide reliable insights into the motifs and their interactions that neural networks have learned from biological data.

## Supplementary Material

btae262_Supplementary_Data
