# Supplementary material for: Unveil cis-acting combinatorial mRNA motifs by interpreting deep neural network
Source: Bioinformatics. 2024 Jun 28;40(Suppl 1):i381–9. doi: 10.1093/bioinformatics/btae262 (PMC11211823; doi:10.1093/bioinformatics/btae262)
Supplement: btae262_Supplementary_Data [file btae262_supplementary_data.pdf]

Supplementary information

Simulation experiment details

We generated two datasets – one with random sequences and the other with manually designed sequences. ”Random sequence” means that each nucleotide in the sequence is randomly selected from ’A, G, C, T’. ”Designed sequence” means that the sequence always contains a pair of motifs, and their positions or spacing between them are indeterminate, while the remaining nucleotides are randomly selected from ’A, G, C, T’.

The calculation rules for the MRL of a sequence are as follows:

1. The Mean Ribosome Load (MRL) of a blank sequence is 0.
2. There are a total of 12 motifs, and each motif can either increase or decrease the MRL for a certain value.
3. When there are two motifs in a sequence, the formula for calculating the MRL is as follows:

   $$Add(A,B) = A + B \quad Syn(A,B) = A \times B \quad Ant(A,B) = log(A + B)$$

   A and B represent the contributions of motif A and motif B to the MRL. “Add” represents the condition where A and B are independent of each other, while “Syn” and “Ant” indicate the presence of synergistic or antagonistic effects between A and B, respectively.
4. There are a total of 36 motif combinations, among which 3 motif pairs exhibit antagonistic effects, 3 motif pairs exhibit synergistic effects, and the other motif pairs exhibit additive effects. In addition, the distance between motifs exhibiting interaction must be within 5 bp.
5. A sequence can have a maximum of two motifs.

For the designed dataset, each sequence is composed of two motifs which are randomly selected from our motif library. In contrast, for the random dataset, the sequences are generated randomly, excluding the sequences containing more than two motifs. The MRL is calculated based on the rules previously discussed. To simulate biological scenarios, a Gaussian noise term is incorporated.

The model architecture used in this simulation experiments is the same as the MRL predictor, which is a hybrid model comprising a 3-layer CNN and GRU. The model’s performance on random dataset and designed dataset is listed below:

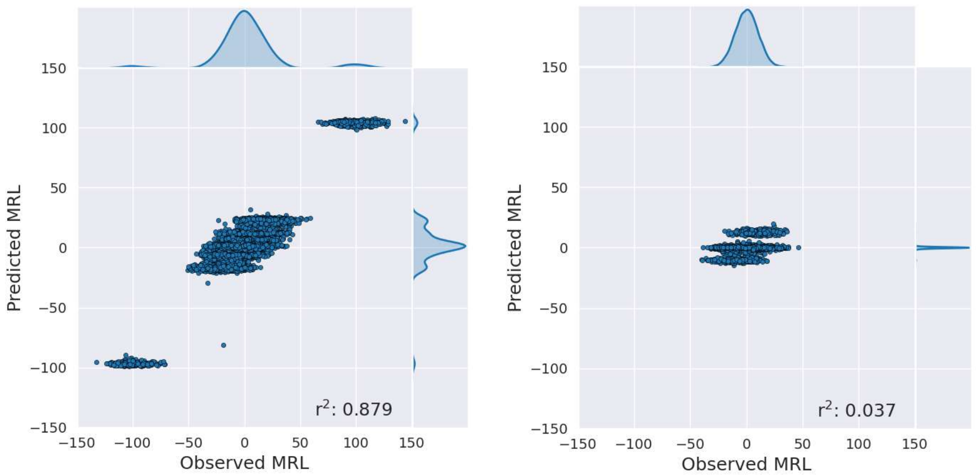

Fig1.The performance of models trained on designed dataset(left) and random dataset(right)

Motifs discovered by NeuronMotif

Table1 The query motifs discovered from Half-life predictor

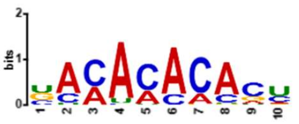 (HNRNPL)	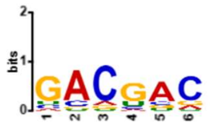 (RBM45)	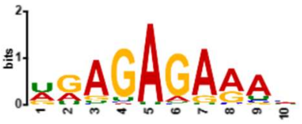 (SRSF10)
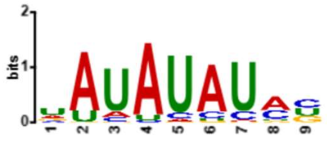 (RBMS3)	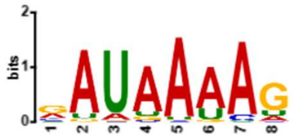 (KHDRBS1)	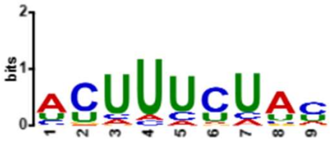 (PTBP1)
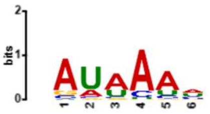 (KHDRBS2)	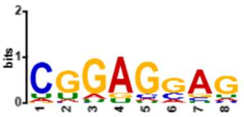 (SRSF1)	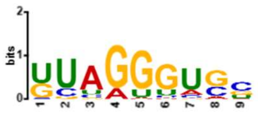 (HNRNPA1L2)
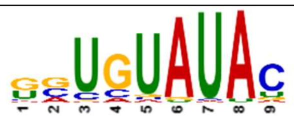 hsa-miR-4789-5p	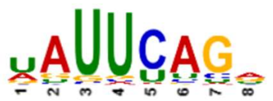 hsa-miR-4427	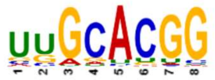 hsa-miR-1973
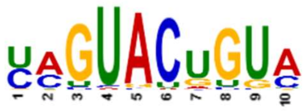 hsa-miR-101-3p	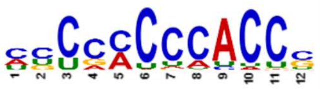 hsa-miR-6769b-5p	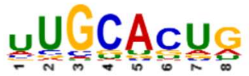 hsa-miR-4295
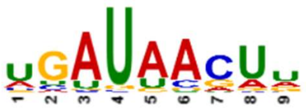 hsa-miR-101-5p	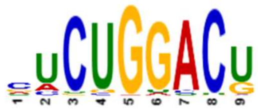 hsa-miR-198	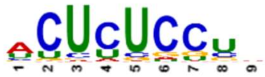 hsa-miR-5192
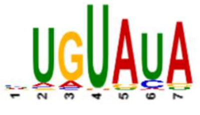 hsa-let-7f-2-3p	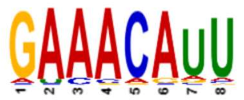 hsa-miR-33a-3p	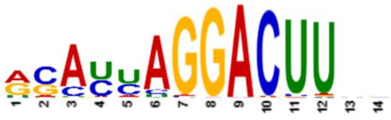 hsa-miR-6838-3p

Table2 The query motifs discovered from MRL predictor(nonAUG)

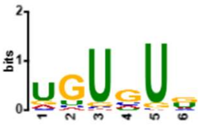 (BRUNOL4)	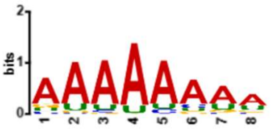 (PABPC4)	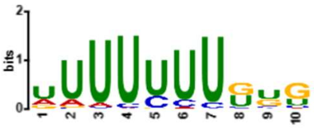 (RALY)
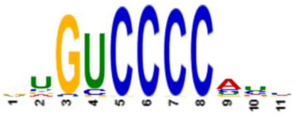 hsa-miR-3191-3p	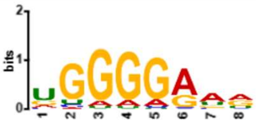 (ESRP2)	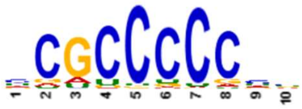 hsa-miR-3196
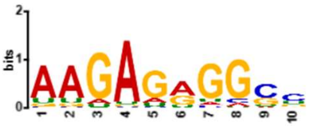 (SRSF10)	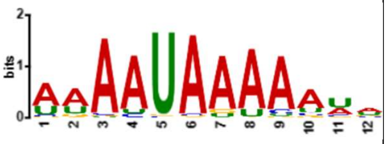 (KHDRBS1)	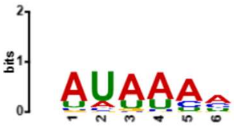 (KHDRBS2)
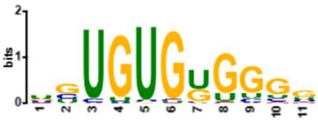 (BRUNOL6)	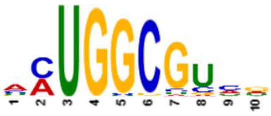 hsa-miR-122-3p	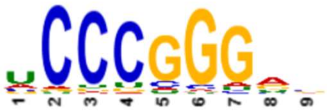 hsa-miR-1247-3p
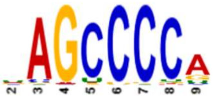 hsa-miR-4283	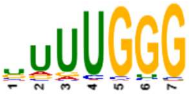 hsa-miR-186-3p	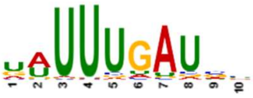 hsa-miR-3671
